# Tracking the Evolution of Transiently Transfected Individual Cells in a Microfluidic Platform

**DOI:** 10.1038/s41598-018-19483-y

**Published:** 2018-01-19

**Authors:** Micaela Tamara Vitor, Sébastien Sart, Antoine Barizien, Lucimara Gaziola De La Torre, Charles N. Baroud

**Affiliations:** 1 LadHyX and Department of Mechanics, Ecole Polytechnique, 91128 Palaiseau, France; 2 School of Chemical Engineering, Department of Bioprocesses and Materials Engineering, University of Campinas (Unicamp), Av. Albert Einstein, 500, Campinas, SP 13083-852 Brazil; 3 Institut Pasteur, Physical Microfluidics and Bioengineering laboratory, Département Génomes et Génétique, 25-28 rue du Dr. Roux, 75015 Paris, France

## Abstract

Transient gene expression (TGE) technology enables the rapid production of large amount of recombinant proteins, without the need of fastidious screening of the producing cells required for stable transfection (ST). However, several barriers must be overcome before reaching the production yields using ST. For optimizing the production yields from suspended cells using TGE, a better understanding of the transfection conditions at the single cell level are required. In this study, a universal droplet microfluidic platform was used to assess the heterogeneities of CHO-S population transiently transfected with cationic liposomes (CL) (lipoplexes) complexed with GFP-coding plasmid DNA (pDNA). A single cell analysis of GFP production kinetics revealed the presence of a subpopulation producing higher levels of GFP compared with the main population. The size of high producing (HP) cells, their relative abundance, and their specific productivity were dependent on the charge and the pDNA content of the different lipoplexes: HPs showed increased cell size in comparison to the average population, lipoplexes with positive charge produced more HPs, and lipoplexes carrying a larger amount of pDNA yielded a higher specific productivity of HPs. This study demonstrates the potential for time-resolved single-cell measurements to explain population dynamics from a microscopic point of view.

## Introduction

In the past decades, Chinese hamster ovary (CHO) cells have emerged as one of the most powerful tools for the production of recombinant therapeutic proteins. CHO cells can secrete very high recombinant product yields, whose glycosilation profile promotes their efficient bioactivity. Moreover, suspended CHO cells have been successfully adapted for expansion in serum free media and for large-scale culture in stirred tank bioreactors^1,2^.

The first step to achieve the production of recombinant proteins by CHO cells requires their genetic engineering. While CHO cells can be easily transfected, current protocols require transgene integration in the host genome, which is usually followed by lengthy procedure of clonal selection and adaptation to large-scale and animal protein free culture conditions^3^. Contrasting with these labor-intensive procedures of stable transfection (ST), transient gene expression (TGE) technology enables the rapid production of large amounts of recombinant proteins, without the need of fastidious screening of the producing cells. Moreover, no adaptation to new culture conditions is required^4^. However, several barriers need to be overcome before reaching the production yields achievable by ST. Among them, because of the episomal location of transgene, exogenous DNA is lost after several rounds of cell division. As a consequence, the production occurs for significantly shorter time period than ST. In addition, the limited control over DNA delivery induces a large heterogeneity in the production rate of individual cells^5^. Consequently, in view of optimizing the production yields, a better understanding and control of the transfection conditions at single cell level using TGE are critically required.

Several carrier systems, such as viral and nonviral vectors, are currently used for the delivery of recombinant nucleic acids into producing cells^6^. While nonviral systems complexed with nucleic acids (e.g. lipoplexes and polyplexes) show significantly lower transfection efficiency, they demonstrate biological inertness, improved safety and they can be easily produced at large-scale, in contrast to viral systems. Consequently, current research efforts are directed to increase the efficiency of non-viral vectors for DNA delivery, by better understanding the mechanism of lipoplex uptake and internalization^7^.

On the other hand, the recent advances in microfluidics have created exciting prospects for gene delivery and therapy. The controlled hydrodynamics within microfluidic systems enables precise control of parameters involved in gene transfection, together with a significant reduction of the volumes of reagents^8^. More specifically, droplet microfluidics has enabled the development of new tools for cell manipulation, such as the encapsulation of individual cells, the biological compartmentalization etc.^9,10^. The encapsulation within droplets can offer many benefits, for instance the ability to accumulate secreted molecules within the small droplet volume, which would lead to high local concentrations that can easily be detected^11^. Nevertheless droplets suffer from to the difficulty to vary their content in time or to track the evolution within an individual droplet. This has limited their application to the simple demonstration non-viral transfection within droplets^12^, leaving a very large part of the potential of droplet encapsulation unexplored.

In this context, the current study tracks the response of a population of CHO-S cells (recombinant Chinese hamster ovary cells suspension culture) after transient transfection with lipoplexes containing different amounts of pDNA. This is performed by building on the recently reported microfluidic platform that allows individual cells to be maintained in culture, transfected, and observed in a single integrated microfluidic device^13^. This protocol then yields measurements of the time-evolution of each cell under dynamically controlled conditions. Below we discuss first the production of the lipoplexes at different molar charge ratios from cationic lipids and negative charge from nucleic acids. This is followed by a description of the integrated cell culture and transfection chip then by an evaluation of the transfection results on the single-cell level. We show that different sub-populations can be distinguised and quantified in this way, with transfection and production efficiencies that depend on the lipoplex charge ratio.

## Results

TGE in suspension enables the production of high recombinant protein yields, without the need of lengthy procedures of clonal selection. However, there have been few investigations of the contribution of individual cells on the rate of recombinant protein production of the global population, while cultivated in suspension. Below we investigate the heterogeneities of GFP expression in a CHO-S population transiently transfected with different types of lipoplexes made with different molar charge ratios (R_+/−_).

### Characterization of the Physico-Chemical Properties of Liposomes and Lipoplexes

**Figure 1:**
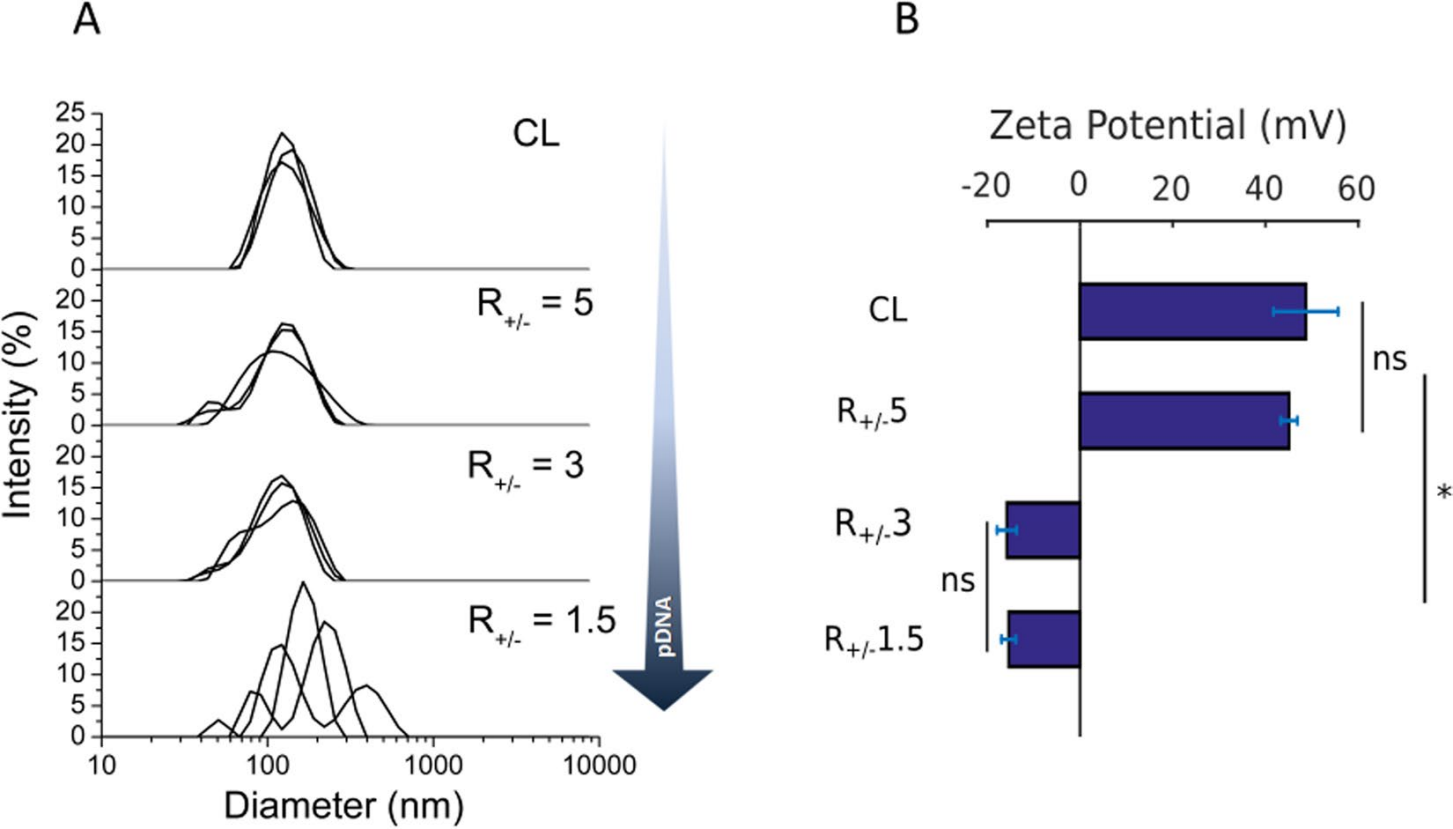
Physico-chemical properties of the cationic liposomes (EPC/DOTAP/DOPE) and the R_+/−_ 5, R_+/−_ 3 and R_+/−_ 1.5 lipoplexes. (**A**) Intensity-weighted size distribution. Each solid line represents one independent size measurement. (**B**) Zeta potential of the cationic liposome and the R_+/−_ 5, R_+/−_ 3 and R_+/−_ 1.5 lipoplexes. *p-value < 0.05; n.s. = not significant (Kruskall-Wallis test). Results show mean ± S.D., N = 3.

The size, polydispersity index (PdI), and zeta potential (ζ) of the cationic liposomes (EPC/DOTAP/DOPE, CL) and the different lipoplexes (R_+/−_ 1.5, R_+/−_ 3, R_+/−_ 5) (Supplementary Information 1) were analyzed by dynamic light scattering (Fig. 1). The average diameter of CL was of 113 ± 1.5 nm. The diameter of the liposome showed a monomodal distribution, as shown by a single peak around 110 nm (Fig. 1A). The polydispersity index (PdI) of the CL was of 0.19 ± 0.01. For lipoplexes, the average size was 108 ± 1.2, 106 ± 1.4 and 158 ± 9.7 nm, for R_+/−_ 5, R_+/−_ 3 and R_+/−_ 1.5, respectively; and the PdIs ranged from 0.15 to 0.21. Increasing the pDNA content into the liposomes (*i.e*. decreasing R_+/−_ values from 5 to 1.5) increased the size dispersion (Fig. 1A). As previously reported, the number of multilayered lipoplexes increases with pDNA copy number. Indeed, the isoelectric point of the EPC/DOTAP/DOPE lipoplexes is around R_+/−_ 1.8. Beyond this ratio, pDNA is in excess, which leads to the formation of a fraction of lipoplexes aggregates, which can fuse to form multiple lipid layers^14^.

The electric charge of liposomes and the different lipoplexes was analyzed by measuring their zeta potential (ζ). The CL presented cationic characteristic, ζ = 48.7 ± 13.9 mV (Fig. 1B). The net charge of R_+/−_ 5 lipoplexes was similar to the empty liposomes (ζ = 45.1 ± 3.6 mV). The complexation with higher pDNA copy number significantly (p-value = 0.04) increased the electronegativity of R_+/−_ 3 and R_+/−_ 1.5 lipoplexes (*i.e*. − 15.8 ± 4.2 mV and − 15.4 ± 3.1 mV, respectively) (Fig. 1B). In contrast, previous studies measured a positive zeta potential for R_+/−_ 3 and R_+/−_ 1.5 lipoplexes, prepared with the same EPC/DOTAP/DOPE mixtures^14,15^. The differences can be related to different solvent used for liposome synthesis and pDNA complexation: in this study, DEPC-treated water was used as a diluent, while PBS was used in the preceding studies.

### Single CHO-S Cell Trapping and Transfection on Chip

The chip for the cell culture experiments consisted of a high aspect ratio chamber equipped with 1495 capillary “anchors”, i.e. regions with an increased depth compared with the rest of the device^16–18^. The protocol began by filling the entire chip with the oil, into which an aqueous phase containing CHO-S cells, liquid agarose and culture medium was injected. The capillary anchors provided low confinement areas for the aqueous phase, contrasting with its higher confinement in the culture chamber. Then aqueous droplets were broken off inside each anchor by flowing oil again into the chamber containing the aqueous phase. As reported previously^18^, the droplet size was determined primarily by the anchor geometry and was relatively insensitive to the physical properties of the fluids or the flow rates. Consequently, the procedure yielded 1495 monodisperse aqueous droplets having a volume 2 nL, each trapped in an anchor (Supplementary Figure S1).

**Figure 2:**
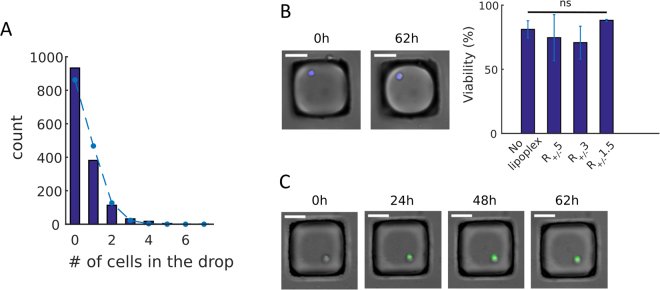
Culture and transfection of CHO-S on chip. (**A**) Distribution of the number of cells per droplet, fitted with a Poisson curve (λ = 0.45) (dashed line). (**B**) Representative image of a Live/Dead stained cell at t = 0 and 62 hours in culture and CHO-S viability post transfection with R_+/−_ 5, R_+/−_ 3 and R_+/−_ 1.5 lipoplexes. *p-value < 0.05, n.s. = not significant (Kruskall-Wallis test). Results show mean ± mean ± M.D. (mean deviation), N = 2. (**C**) Representative image of GFP producing CHO-S at t = 0, 24, 48 and 62 hours in culture. White scale bars are 50 µm.

After trapping, the distribution of CHO-S cells inside the anchors followed a Poisson law, indicating that the cells were distributed randomly and did not clustered during the loading (Fig. 2A). Under this loading condition, about 30% of the droplets contained only one single cell, which were then analyzed after transfection.

As the CHO-S cells were encapsulated in agarose, its gelation mechanically retained the cells in the anchors, which allowed performing several rounds of phase exchange (*i.e*. oil-to-aqueous and aqueous-to-oil). This phase exchange was necessary to wash the droplets, bring in fresh culture medium or staining molecules, and then re-isolate the droplets from each other. Thus, the protocol resulted in the isolation of single CHO-S cells and it enabled long-term culture (at least up to 62 hours) with a tracking of individual cells. By the end to the culture period, the isolated single CHO-S cells (in the absence of transfection) remained viable on-chip (viability = 81 ± 13%) (Fig. 2B).

Next, the platform was used to study the CHO-S productivity of GFP by transient transfection using lipoplexes with different molar charge ratio. For this purpose, the aqueous phase containing CHO-S cells, liquid agarose and culture medium was supplemented with the CL complexed with pDNA. In the total process (incubation + solidification of the gel + chip loading), CHO-S cells remained in contact with lipoplexes for 4 hours in the anchors. Then, phase exchange was performed to wash the excess of lipoplexes from the chip. The potential toxicity of the lipoplexes was examined by Live/Dead staining and the evolution of GFP production was monitored by time lapse, starting immediately after the washing of the excess of lipoplexes. The cells remained viable after transfection and long-term culture (Fig. 2B**)**, whatever the type of lipoplexes (75 ± 36% for R_+/−_ 5, 71 ± 25% for R_+/−_ 3 and 88 ± 1% for R_+/−_ 1.5). In addition, the gradual increase in GFP signal indicated the efficacy of the transfection under the different conditions tested (Fig. 2C).

### Kinetics of GFP Production at Single CHO-S Level Revealed Distinct Populations

**Figure 3:**
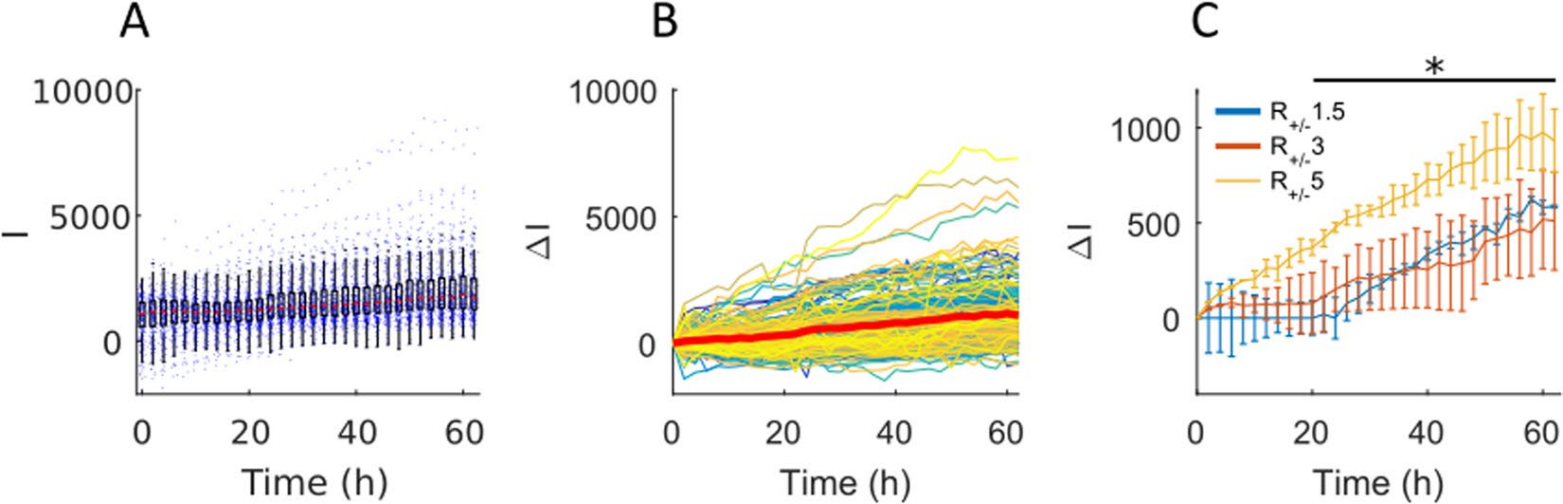
Population level analysis of GFP production post transfection. (**A**) Representative time evolution of raw fluorescent intensity of GFP, I(t), for cells transfected with the lipoplex R_+/−_ 5. (**B**) Representative time evolution of GFP fluorescent intensity variation, ΔI. The bold red line represents the average ΔI of the whole population. (**C**) Time evolution of ΔI for cells transfected with lipoplexes R_+/−_ 1.5, R_+/−_ 3 and R_+/−_ 5. Results show mean ± M.D. (mean deviation), N = 2, (R_+/−_ 1.5: n_cells_ chip1 = 189 and n_cells_ chip2 = 320 cells; R_+/−_ 3: n_cells_ chip1 = 331 and n_cells_ chip2 = 223 cells; R_+/−_ 5: n_cells_ chip1 = 163 and n_cells_ chip2 = 223 cells). *p-value < 0.05 (Kruskall-Wallis test).

The kinetics of the GFP increase were tracked by taking an image of the whole chip every two hours. This imaging, associated with the image analysis, provided a measure of the evolution of GFP production the scale of the whole population of cells. As shown in Fig. 3A for the case of the R_+/−_ 5 lipoplexes, the values of the GFP signal obtained in this manner were similar to those that could be obtained from flow cytometry: they could yield a mean and distribution of values at any given time point.

This point of view however hides a more interesting dynamical measurement, which is the time evolution of each single cell. Since the cells are identified individually, this time evolution can be obtained by defining Δ**I** = I(t) − I_0_, where I_0_ is the fluorescence intensity at the initial instant and I(t) is the value at every instant. The resulting measurements for the R_+/−_ 5 conditions are shown in Fig. 3B and similar trends are observed for R_+/−_ 3 and R_+/−_ 1.5 (Supplementary Figure S2). A first observation is that most individual curves increase linearly in time (r^2^ > 0.9, Supplementary Figure S3), albeit at a different slope for each cell. Moreover, most of the cells follow a steadily increasing trend in GFP signal, which is distributed around the mean curve (shown as the bold red line, Fig. 3B).

At this stage the mean GFP production by the population can be compared for the different lipoplexes, as shown in (Fig. 3C). The comparison shows that transfection with the R_+/−_ 5 lipoplexes leads to higher GFP production (1.5 fold) in comparison with the R_+/−_ 3 and R_+/−_ 1.5 conditions. This result indicates that the R_+/−_ 5 lipoplexes, which presented positive charge, were more efficient at transfecting the general population than R_+/−_ 3 and 1.5 with negative charge.

**Figure 4:**
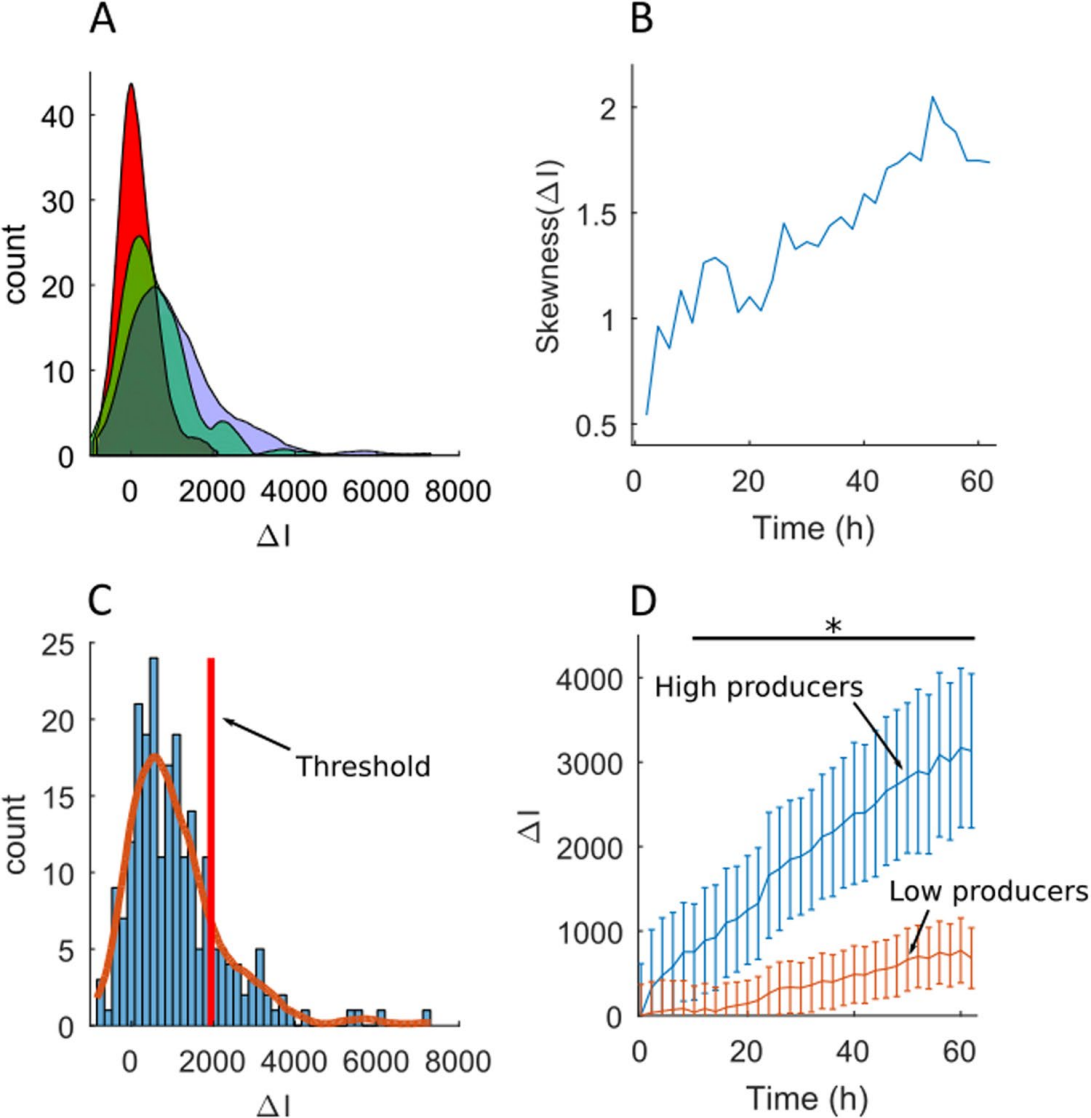
Single-cell level analysis of GFP production post transfection. (**A**) Distribution of ΔI values for cells transfected with R_+/−_ 5 lipoplexes, at t = 12 hours (red curve), t = 32 hours (green curve) and t = 62 hours (blue curve). (**B**) Time evolution of the skewness (third moment) of the ΔI distribution. (**C**) Representative example of the threshold calculation, which discriminates the high producers (HPs) from the rest of the population (R_+/−_ 5 lipoplexes, at 62 hours). (**D**) Time evolution of ΔI of HPs (blue line) and the rest of the population (red line). Results show mean ± mean ± M.D. (mean deviation), N = 2, (R_+/−_ 1.5: n_cells_ chip1 = 189 (9 HPs) and n_cells_ chip2 = 320 cells (14 HPs); R_+/−_ 3: n_cells_ chip1 = 331 (29 HPs) and n_cells_ chip2 = 223 cells (16 HPs); R_+/−_ 5: n_cells_ chip1 = 163 (17 HPs) and n_cells_ chip2 = 223 cells (38 HPs). *p-value < 0.05 (Wilcoxon rank sum test).

Figure 3B however also shows that some cells depart significantly from the mean behavior, by displaying a far greater value of ΔI than the population mean (p-value < 0.05 after t = 18 h). This becomes more evident when plotting the histogram of ΔI as a function of time (Fig. 4A). These distributions display a well-defined peak value but with a tail towards the high values of ΔI that grows with time, indicating the emergence of a subpopulation of high GFP producing cells. A measure of the asymmetry in the distribution is given by its third moment: its skewness. A positive value of the skewness indicates that the distribution extends more to the right of the mean than to the left. In all of our measurements, the skewness starts out small and increases with time (Fig. 4B). This robust behavior is a signature of the existence of the subpopulation that extends towards the large values of ΔI.

The population could therefore be divided into two sub-groups by observing the distribution of ΔI at the final time point (Fig. 4C**)** and defining a threshold value of ΔI for each experiment, based on the asymmetry of the final distribution. Cells whose value of ΔI was above this threshold were labeled as high producers (HPs), while those with ΔI value below the threshold were low producers (LPs) such that the evolution of each sub-population could be studied independently (Fig. 4D). Of note, the HPs were evenly distributed within the chip (Supplementary Figure S4).

### Characterization of the High GFP Producers

**Figure 5:**
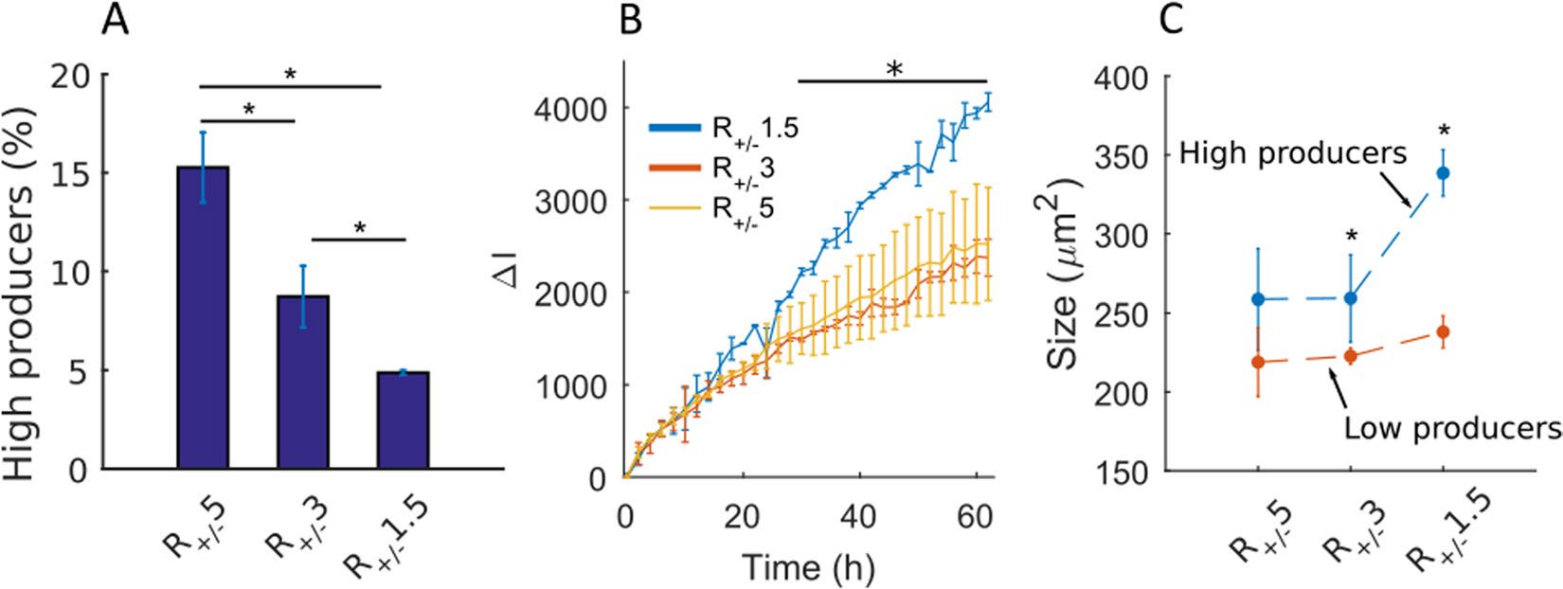
Characterization of high GFP producers (HPs) after transfection with R_+/−_ 5, R_+/−_ 3 and R_+/−_ 1.5 lipoplexes. (**A**) Percentage of HPs after transfection with R_+/−_ 5, R_+/−_ 3 and R_+/−_ 1.5 lipoplexes. (**B**) Time evolution of ΔI of HPs after transfection with R_+/−_ 5, R_+/−_ 3 and R_+/−_ 1.5 lipoplexes. (**C**) Cell area of HPs after transfection with R_+/−_ 5, R_+/−_ 3 and R_+/−_ 1.5 lipoplexes. Results show mean ± M.D. *p-value < 0.05 (Wilcoxon rank sum test, panels A and C; Kruskal-Wallis, panel B). R_+/−_ 5: n_cells_ chip1 = 163 (17 HPs) and n_cells_ chip2 = 223 cells (38 HPs).

The distinction between low producers and high producers could be made whatever the type of lipoplexes used for recombinant DNA delivery, although the details varied between different conditions. The influence of the lipoplexes at different molar charge ratios was therefore investigated, first on the relative abundance of HPs and then on their specific productivity. A higher percentage of HPs was found when using lipoplexes R_+/−_ 5 (15.27% ± 1.77), compared to R_+/−_ 3 (8.72% ± 1.55) and R_+/−_ 1.5 (4.88% ± 0.12) (Fig. 5A). Conversely, the opposite trend was found when measuring the specific GFP productivity of HPs, defined as the average value of ΔI expressed only by HPs (Fig. 5B). Here the GFP productivity for R_+/−_ 1.5 was found to be about 1.5 times higher than the specific productivity for R_+/−_ 5 and R_+/−_ 3, which were equal to each other (Fig. 5B).

These two results suggest that the higher productivity of CHO-S population transfected with R_+/−_ 5 (Fig. 3C) was mainly due to higher percentage of HPs within the whole population (Fig. 5A). In contrast, the number of HP cells in the R_+/−_ 1.5 condition was smaller but each of these cells produced a stronger level of GFP than the other two conditions. But despite the higher specific productivity, the contribution of the HPs in R_+/−_ 1.5 condition (Fig. 5B) was not sufficient to reach the same total level of GFP production than for R_+/−_ 5.

In order to test the underlying reasons for the observed differences in GFP expression, we looked for different morphological differences between low producers and high producers. A difference in the cell size at the beginning of the experiment was found between the two sub-populations for all three lipoplexes. Indeed, the size distributions were different for the two subpopulations (Supplementary Figure S5) and the average HPs area was at least 15% higher than LPs whatever the type of lipoplexes tested (Fig. 5C). In turn, the cells transfected with R_+/−_ 1.5 and R_+/−_ 3 lipoplexes had HPs significantly bigger than LPs (p-value < 0.01). Moreover, the initial area of HPs transfected with R_+/−_ 1.5 was significantly larger than those with R_+/−_ 5 and 3 (Fig. 5C). Thus, the cell size emerged as an important parameter leading to the emergence of HPs in the transfected CHO-S population.

## Discussion

The mechansisms of nucleic acids transfection using non viral delivery systems has been well characterized, and mathematical models can now accurately predict the kinetics of protein production for adherent cultures in 2D^19–24^. Indeed, recent single cell analyses of the recombinant protein production after TGE have revealed several levels of stochasticity in the transfection process in 2D, due to the nanoparticle size distritbution, the cell cycle distribution, the cell’s membrane charge, the trafficking of the nucleic acids, etc.^19–24^. These studies were possible by taking advantage of the adhesion of the cells to the solid substrate, which allows them to be subjected to different solutions and observed over time.

By contrast, there have been far fewer ingestivations on the heterogenity of the cell responses when cultivated and transfected in suspension, even though the selection of producing clones in suspension cultures is a critical step for industrial scale protein production in stirred bioreactors. The limited number of such studies is due in part to the complexity of manipulating suspended cells while observing their evolution in time. Nevertheless, novel technologies based on fluorescent imaging for characterizing (e.g. size, shape, kinetics protein expression etc.) and recovering (e.g. automated cell pickers) HPs cultivated in semi-solid media (e.g. ClonePix^TM^, Cell-Celetor^TM^) have led to significant improvement for isolating highly productive clones, in comparison to conventional limiting dilution cloning^25^. However, beside their relative high costs, these new tools suffer from a lack of cell biological isolation, leading to potential paracrine interference in the dynamcics of recombinant production of indivudualized cells. In addition, the cell encapsulation within thick hydrogels using ClonePix^TM^ may locally expose the cells to hetereogeneous mechanical cues^26^ and diffusion limitations^27^, which may perturb single cell production as well.

In this context, developing more robust investigation techniques will provide a better understanding of the kinetics of protein production of individual cells after TGE. When combined with non-invasive selection of the HP cells, this would lead to substantial bioprocess improvement for increasing the yield of biomolecules. Droplet microfluidics provides a cost-effective approach to encapsulate and manipulate individual cells at microscale^28^, though the technique suffers from two major drawbacks in this context. First, traditional methods do not allow individual droplets to be followed in time, since most methods require the cell-containing droplets to be produced in series and stored outside the microfluidic device^28^. Their contents are then generally detected in a manner similar to flow cytometry. Second, although merging droplets together allows the addition of solutions into a pre-formed droplet^28^, removing chemical species from droplets is not generally possible. As such, transient transfection protocols cannot be performed in traditional droplet microfluidics.

In this manuscript we demonstrate, for the first time, the transfection and quantitative analysis of non-adherent mammalian cells in a microfluidic platform. The platform is built around an integrated device to transiently subject the cells to a chemical or biological stimulus and to culture them for several days while observing the time evolution of each cell. In contrast with flow cytometry, the ability to follow the evolution of the signal from each cell vastly improves the signal-to-noise ratio in the data, by transforming absolute measurements into comparative measurements.

The unique window provided by the microfluidic platform to observe the kinetics of recombinant proteins at single cell level revealed the heterogeneity of a CHO-S population post transfection. The population contained a discrete sub-population (HPs) producing higher levels of GFP and having larger size in comparision with the rest of the population. These results were consistent with the observations previously reported in 2D cultures, in which the specific productivity was usually higher in the G2/M phase of the cell cycle^5,29^. During this phase more rRNAs are available for recombinant protein translation and the loss of nuclear membrane integrity facilitates the plasmid internalization through the endosomal pathway^30^. In addition, as the cytoplasmic membrane is extending prior to mitosis, the decrease of its tension facilitates the endocytosis of nanoparticles^31^. In the same vein, the topography-induced increase of the cell size (e.g. using micropillars) in 2D also resulted in a significant increased the transfection efficiency^32^.

We also explored the effect of different types of lipoplexes on the recombinant protein productions in our cells. At the population level, our results showed that transfection with more cationic liposomes (*i.e*. R_+/−_ 5) led  to higher GFP production, which was consistent with previous observations on 2D cultures where pDNA transfection was found to be enhanced by charge interactions between the lipoplexes and membranes^15,33^. In addition, our single cell level analysis demonstrated significantly higher percentage and larger HPs when transfected with highly cationic liposomes. In contrast, less cationic lipoplexes (i.e. containing more pDNA) promoted higher GFP specific productivity of the HPs than the cells transfected with more positively charged lipoplexes. Indeed, at low charge ratios, the lipoplexes contain high pDNA copies^34^, but their capability to form cell-complexes interactions is reduced^15,33^.

Beyond the current study, the microfluidic platform that we have demonstrated can be extended in several different directions. First of all, it can directly be applied for secreted molecules, by taking advantage of the encapsulation within the microfluidic droplets, which allow more rapid accumulation of recombinant proteins^35^. In this context the encapsulation within the droplet’s small volume will allow the detection of even small amounts of proteins. Beyond on-chip culture, individual droplets could be selectively recovered from the chip^13^. Such a recovery will allow specific cells (*e.g*. the highest producers) to be extracted and further cultured off-chip, in view of their expansion in macroscopic bioreactors^4^, or to be further analyzed. Finally, the same physical approaches and analyses can be used for other cellular assays, for example for observing the heterogeneity of cellular responses in a drug screening, measuring fermentation efficiency of yeast cells, or to observe cell-cell interactions. As such, the current study demonstrates a first application that can be followed by many more.

## Methods

### Microfabrication

Standard dry soft lithography was used for the fabrication of all microfluidic devices, as previously described^36^. Cationic liposomes (CL) were produced in a hydrodynamic focusing microfluidic device^37^. The cross-junction design has a rectangular cross-section of depth 100 μm and height 135 μm.

For the cell culture chip, the design followed the chip described by Amselem *et al*.^13^. The chip consists in a 2D chamber (Supplementary Figure S1) with two inlets (1 and 2) and one outlet (3). The chamber has a base height h_1_ = 35 μm and it is patterned with anchors whose height is 100 µm, giving a total height h = 135 μm (Supplementary Figure S1)^13^.

### Production and Characterization of CL and their Complexes

Cationic liposomes were formed along the main channel of cross-junction microfluidic device. EPC (egg phosphatidylcholine), DOTAP (1,2-dioleoyl-3-trimethylammonium-propane) and DOPE (1,2-dioleoyl-sn-glycero-3-phosphoethanolamine) (50/25/25% molar) all from (Lipoid, Germany) were dispersed in anhydrous ethanol to achieve 25 mM of total lipid concentration^38^. The lipid dispersion was injected into the center flow at 11 μL min^−1^. Simultaneously, two streams of DEPC-treated water (Life Technologies, USA) were injected at 55 μL min^−1^ into the two lateral sides. Liposome samples were collected from the exit and left for at least 4 hours at 4 °C.

For lipoplex formation, the CL and pDNA pMAX GFP (Lonza, USA) were mixed by vortexing for 30 seconds. Several molar charge ratios of cationic liposomes/pDNA were used to produce lipoplexes at different DNA loading, characterized by the ratio (R_+/−_) between the positive charge from cationic lipids and negative charge from pDNA. Cells were transfected with several types of lipoplexes, which were prepared by mixing 7 × 10^−2^ µg pDNA and different volume of the liposomes solution, varying from 2 µL (R_+/−_ 5), 1 µL (R_+/−_ 3) and 0.5 µL (R_+/−_ 1.5), resulting in 4 µL lipoplexe solutions.

The nanoparticles properties, CL and their lipoplexes were diluted in DEPC water. The average hydrodynamic diameter, the size distribution, polydispersity index and zeta potential were measured by dynamic light scattering (the scattering angle was 173°, backscattering) Zetasizer Nano Series, ZEN3600 (Malvern, UK), and analyzed using CONTIN algorithm.

### Cell Culture and Labeling

FreeStyle CHO-S (Life Technologies, USA) cells were cultivated at 37 °C into 25 cm^2^ Ultra-Low Attachment flasks (Corning, USA) in a humidified incubation set up at 8% CO_2_, using FreeStyle CHO Expression Medium supplemented with 8 mM GlutaMAX (Gibco, USA). The cells were passaged every 48–72 hours, by reseeding at a concentration of 1 × 10^5^ cells.mL^−1^.

To monitor the CHO-S behavior in culture, the cells were labeled with CellTracker Red CMTPX Dye (Thermo Scientific, USA), and cells were stained for ReadyProbes Cell Viability Imaging Kit Blue/Red (Thermo Scientific, USA), to measure the viability.

### Cell Encapsulation in Hydrogels

Type IX ultra-low-gelling Agarose (Sigma-Aldrich, USA) was used to provide a mechanical support for the non-adherent cells to keep them stationary. The electroendosmosis (EEO) of the agarose is low (i.e. <0.12), ensuring limited electrostatic interactions between lipoplexes and the hydrogel. The average pore size of the agarose gels is of about 500 nm, significantly larger than the lipoplexes. For the experiments, a 70 µL solution containing 5 × 10^3^ cells in culture medium was mixed with a 3% liquid agarose solution. The concentration of resulting gels was of about 1%. At this concentration, the mechanical properties of the agarose gel enable the cells to be firmly retained in the anchors^13^. However, it was expected to observe a limited cell growth of the agarose encapsulated CHO-S (e.g. using immunofluorescence on chip^39^), due to the low metastatic potential and the low degree of transformation of CHO-S at low passage number^40^.

### Chip Loading and *In-Situ* Transfection

The cell loading was performed using a three-step protocol^13^. (1) The chip is entirely filled with FC-40 (Fluorinert Electronic Liquid FC-40 from 3 M, USA), a fluorinated oil, supplemented with 5 mg/mL a PEGylated surfactant (FluoroSurfactant, RAN Biotechnologies, USA); (2) the FC-40/surfactant flow is stopped and a solution containing 5 × 10^3^ cells (70 µL), 3% w/v liquid agarose (30 µL) and 4 µL of lipoplexes at different molar charge ratio (R_+/−_ 1.5, R_+/−_ 3 and R_+/−_ 5) is flowed at 10 µL min^−1^. The volume of lipoplexes corresponded to approximately 2–10 × 10^7^ particles/μL (Supplementary Information 2); (3) once the chamber is filled the flow of cells/lipoplex solution is stopped and FC-40/RAN inserted at a flow rate increasing from 5 to 50 µL min^−1^ to push the cells/lipoplexes solution towards the exit. This creates droplets containing the cells and about 10^4^ lipoplexes immobilized on each anchor^18^ (Supplementary Information 2). To promote the interaction between the cells and lipoplexes, the chip was placed for 1 hour in the CO_2_ incubator. The chip was then flushed with pure FC-40 at 20 µL min^−1^, in order to wash away the surfactant, after which the agarose was gelled by placing the chip at 4 °C for 15 minutes that allowed there placement of the oil by culture medium, which was flushed into the chip at 20 µL min^−1^ for 10 minutes. Finally, the chip was filled again with FC-40/surfactant, which surrounded the agarose spheres, thus isolating the droplets from each other. The GFP production was tracked by time-lapse microscopy for 62 hours using a fluorescent microscope Eclipse Ti-U (Nikon, Japan), equipped with an EM-CCD camera (Andor Technology, Northern Ireland).

### Image and Data Analysis

To track the recombinant protein production, images were taken every two hours in bright field and in fluorescence to detect CellTracker Red (red channel) and GFP (green channel). GFP production was measured by quantifying the intensity evolution of each detected cell in the green channel. A custom-made MATLAB code was used to process the images. First, the images taken in the red channel (i.e. labeled for CellTracker Red) were thresholded and converted into masks. The objects with a diameter comprised between 10 and 25 µm were considered as cells. The green channel signal intensity (I_cell_) was then quantified for each cell. The green channel signal measured in rest of the anchors was considered as the background (I_back_). The green channel signal per cell (I) was defined as the value of green channel signal in the mask minus the value of green channel signal in the background: I = I_cell_ − I_back_. To better improve the signal to noise ratio, the difference between the fluorescent intensity a defined time point, I(t), and the initial fluorescent intensity value (I_0_) was calculated, as ΔI = I(t) − I_0_.

### Statistical Analysis

Each experiment was conducted at least two times. To assess the statistical significance between the different experimental groups, the data from single cell analysis in each group were compared using Kruskal-Wallis (multiple comparisons) or Wilcoxon rank (paired comparisons) non-parametric tests, which were chosen based on the analysis of the raw data distribution (Anderson-Darling test or the measure of the data distribution skweness). A p-value < 0.05 was considered statistically significant.

### Data availabity statment

The datasets generated during and/or analyzed during the current study are available from the corresponding author on reasonable request.

## Electronic supplementary material

Supplementary Material
