# Supplementary material for: Tracking the Evolution of Transiently Transfected Individual Cells in a Microfluidic Platform
Source: Sci Rep. 2018 Jan 19;8:1225. doi: 10.1038/s41598-018-19483-y (PMC5775383; doi:10.1038/s41598-018-19483-y)

Tracking the Evolution of Transiently Transfected Individual Cells in a Microfluidic Platform

SUPPLEMENTARY MATERIAL

Micaela Tamara Vitor[1,2], Sébastien Sart[1], Antoine Barizien[1],

Lucimara Gaziola De La Torre[2] & Charles Baroud[1*]

[1]LadHyX and Department of Mechanics, Ecole Polytechnique, 91128 Palaiseau, France

[2]School of Chemical Engineering, Department of Bioprocesses and Materials Engineering, University of Campinas (Unicamp), Av. Albert Einstein, 500, Campinas, SP, 13083-852, Brazil

*corresponding author: baroud@ladhyx.polytechnique.fr

SUPPLEMENTARY INFORMATION

1. Estimation of the molar charge ratio of the lipoplexes

The lipid to DNA charge ratio ($R_{+/-}$) is defined as the ratio of cationic charges from head-groups of lipids in the liposomes divided by the number of anionic charges on the pDNA. It is calculated by considering the following[1]:

A single nucleotide ($M_{nucleotides} \approx 330$ g/mol) contains one phosphate residue, and each phosphate diester bond of a single strand DNA backbone carries one electron (i.e. 1 μg of DNA = 3 nmols of phosphate residues). As a consequence, the number of electrons ($N_{electrons}$) carried by a given mass of DNA (m_{DNA}) is defined by:

$$N_{electrons} = n_{nucleotides} = m_{DNA} / M_{nucleotides},$$

where $n_{nucleotides}$ is the number of moles of nucleotides

DOTAP is the only positively charged lipid in the EPC/DOTAP/DOPE complexes, and DOTAP contains one cationic charge per molecules. The number of protons carried by a given quantity of liposomes:

$$N_{protons} = n_{DOTAP} = [DOTAP] \times V,$$

where [DOTAP] is the molar concentration of DOTAP and V the total volume containing the lipoplexes, and n_{DOTAP}, the number of moles of DOTAP.

Consequently, $R_{+/-}$ is calculated as:

$$R_{+/-} = N_{protons} / N_{electrons} = n_{DOTAP} / n_{nucleotides} = ([DOTAP] \times V) / (m_{DNA} / M_{nucleotides})$$

	**$R_{+/-}$ 5**	**$R_{+/-}$ 3**	**$R_{+/-}$ 1.5**
[DOTAP]	0.57 mM	0.57 mM	0.57 mM
V	2 μL	1 μL	0.5 μL
**n_{DOTAP}**	**1.14 nmol**	**0.57 nmol**	**0.28 nmol**
m_{DNA}	7 x 10^{-2} μg	7 x 10^{-2} μg	7 x 10^{-2} μg
$M_{nucleotides}$	330 g/mol	330 g/mol	330 g/mol
**$n_{nucleotides}$**	**0.21 nmol**	**0.21 nmol**	**0.21 nmol**

2. Estimation of the number of bilayered lipoplexes per droplet on chip

The number of lipid per liposomes (N_{Lipids}) for EPC/DOPE/DOTAP (50/25/25% molar) mixtures is about 7.2 x 10^5 lipid molecules / liposome[2]. The number of liposomes in the stock solution is given by:

$$[Liposomes] = (M_{lipids} \times N_A) / (N_{Lipids} \times 1000)$$

$$[Liposomes] = 2 \times 10^{12} \text{ liposomes / mL}$$

where M_{lipids} is the molar concentration of lipids (2.28 mM) ; N_A, the Avogadro number (6.02 x 10^{23}).

	$R_{+/-}$ 5	$R_{+/-}$ 3	$R_{+/-}$ 1.5
Volume used from stock solution to form the lipoplexes (in 40 µL)	2 µL	1 µL	0.5 µL
Number of total lipoplexes	4 x 10^9	2 x 10^9	1 x 10^9
Percentage of bilayered lipoplexes[3]	20%	48%	66%
Number of bilayered lipoplexes per drop (2 nL)	4 x 10^4	5 x 10^4	3 x 10^4

References

1. Rädler, J. O., Koltover, I., Jamieson, A., Salditt, T. & Safinya, C. R. Structure and Interfacial Aspects of Self-Assembled Cationic Lipid−DNA Gene Carrier Complexes. *Langmuir* **14,** 4272–4283 (1998).

2. Torre, L. G., Carneiro, A. L., Rosada, R. S., Silva, C. L. & Santana, M. H. A. A mathematical model describing the kinetic of cationic liposome production from dried lipid films adsorbed in a multitubular system. *Brazilian J. Chem. Eng.* **24,** 477–486 (2007).

3. Balbino, T. A. *et al.* Correlation of the Physicochemical and Structural Properties of pDNA/Cationic Liposome Complexes with Their in Vitro Transfection. *Langmuir* **28,** 11535–11545 (2012).

SUPPLEMENTARY FIGURES

Figure S.1. Chip design and dimensions for the cultivation of CHO-S cells. (A) Large scan of the entire chip. Black scale bar is 1 mm. (B) Chip top view. The chip is equipped with 2 inlets for oil and the aqueous phase and one outlet. The chip is patterned with 1495 capillary anchors. The dimensions of the anchors are: d = 120 µm of side, spaced by δ = 240 µm. (B) Lateral section of the chip. The chamber height (h_1) is 35 µm; the anchor height (h) is 135 µm.

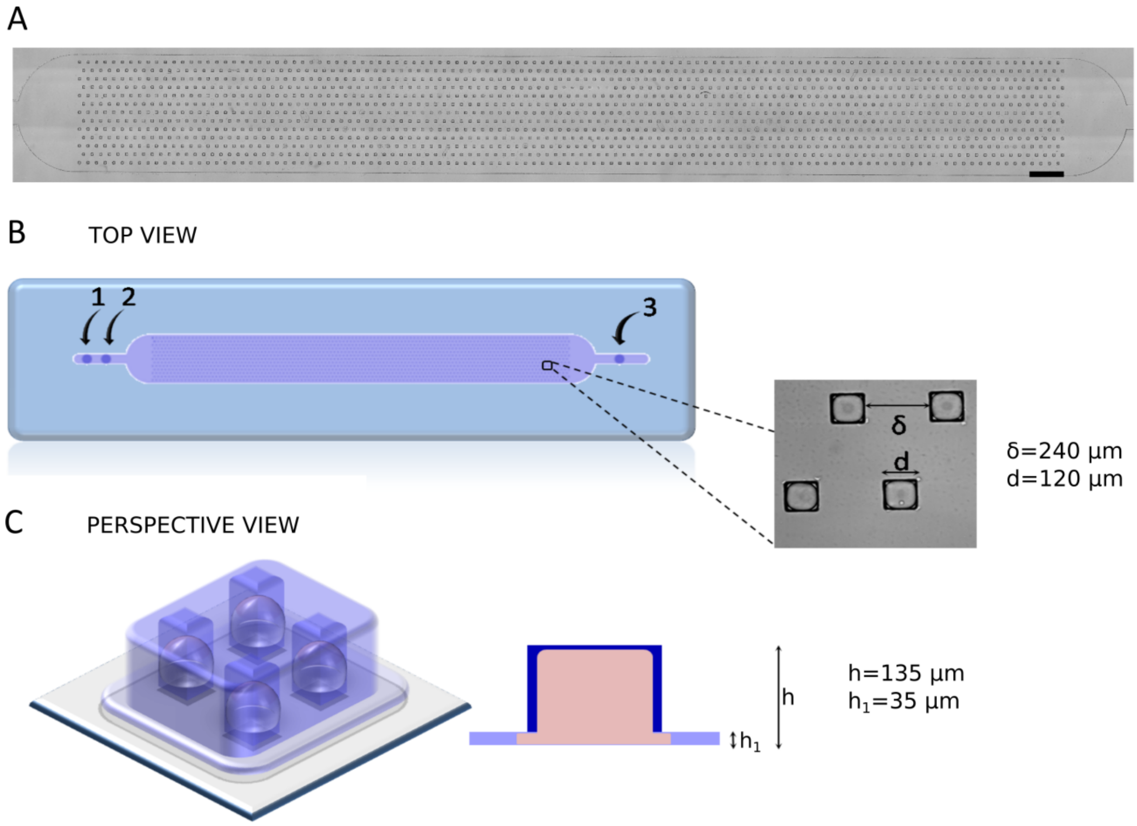

Figure S.2. Population-level analysis of GFP production post transfection with $R_{+/-}$ 3 and $R_{+/-}$ 1.5 lipoplexes. Representative time evolution of raw fluorescent intensity of GFP, I(t), for cells transfected with $R_{+/-}$ 3 (A) and $R_{+/-}$ 1.5 (B) lipoplexes. Representative time evolution of GFP fluorescent intensity variation, ΔI, which is I(t) substracted with I(t) at t= 0 h in culture, for cells transfected with $R_{+/-}$ 3 (C) and $R_{+/-}$ 1.5 (D) lipoplexes. The bold red line shows the average ΔI of the whole population.

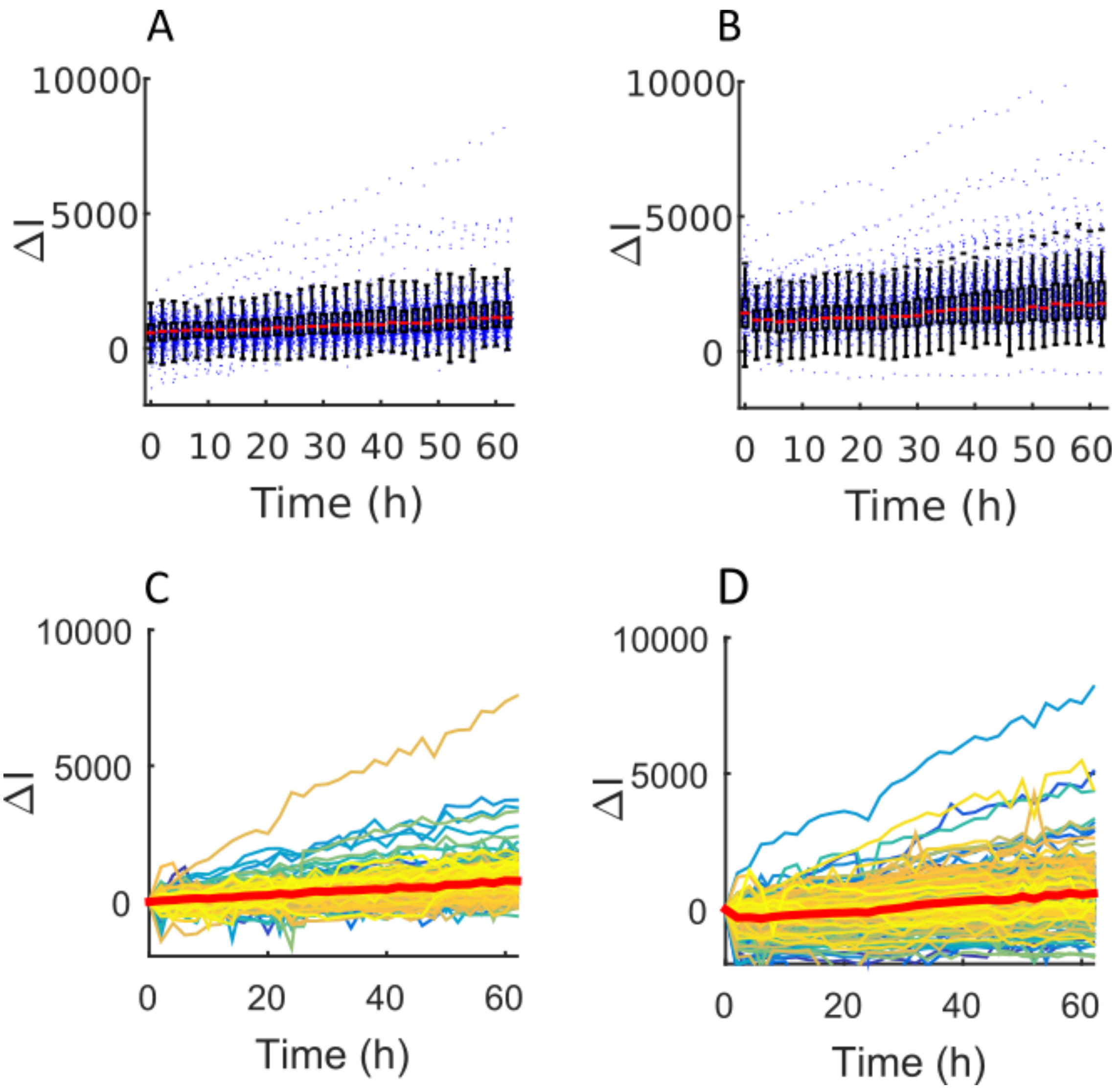

Figure S.3. Distribution of the coefficient of regression (r^2) of the ΔI time evolution curves. Representative r^2 distribution of the ΔI time evolution of the whole population of cells transfected with $R_{+/-}$ 5 lipoplexes (blue columns). The red cure shows the r^2 distribution for the high GFP producers only.

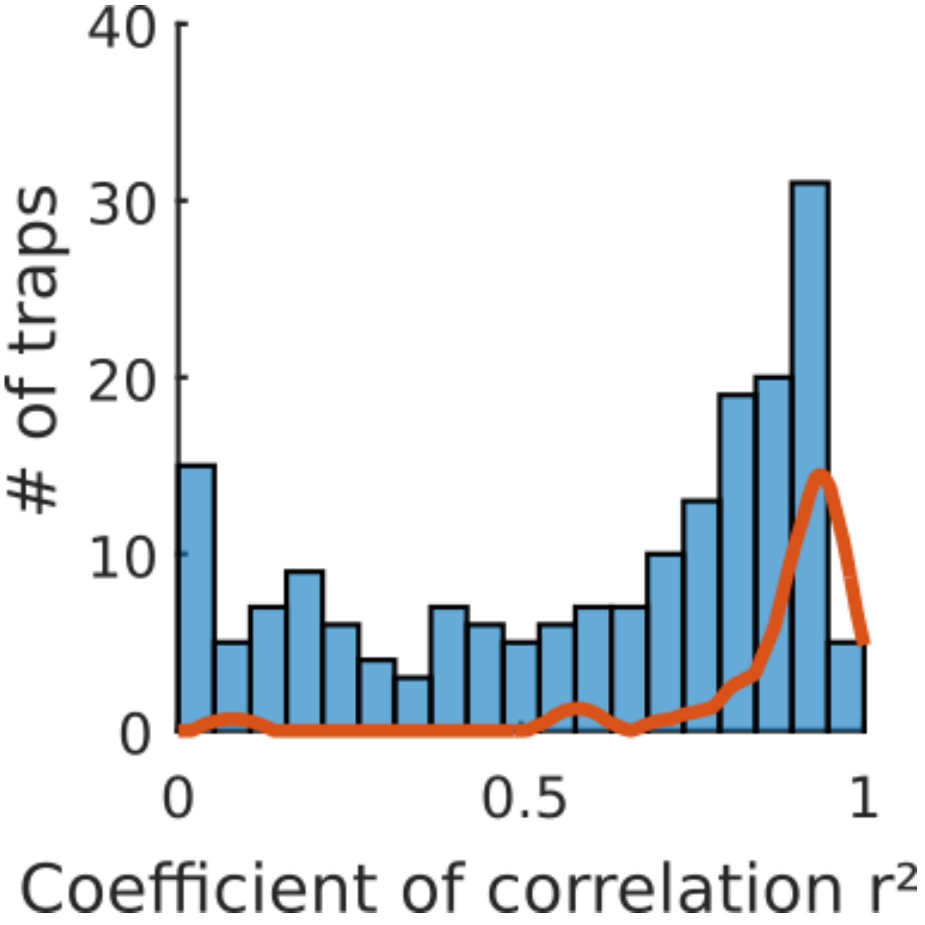

Figure S.4. Spatial distribution of HPs on chip. The HPs are shown as red dots on the chip's map ($R_{+/-}$ 5 lipoplexes).

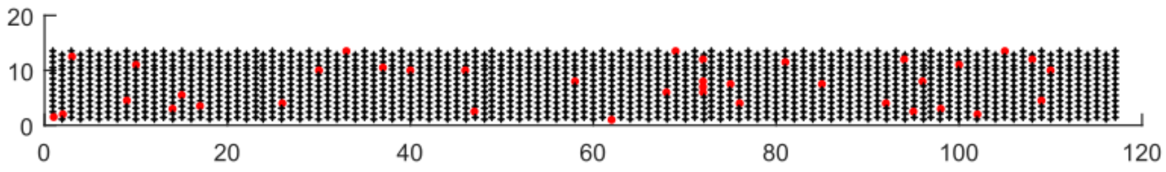

Figure S.5. Size distribution of CHO-S post transfection with $R_{+/-}$ 5 lipoplexes. The size distribution of the whole population is shown in blue bars; the red cure shows the size distribution for the HPs only.

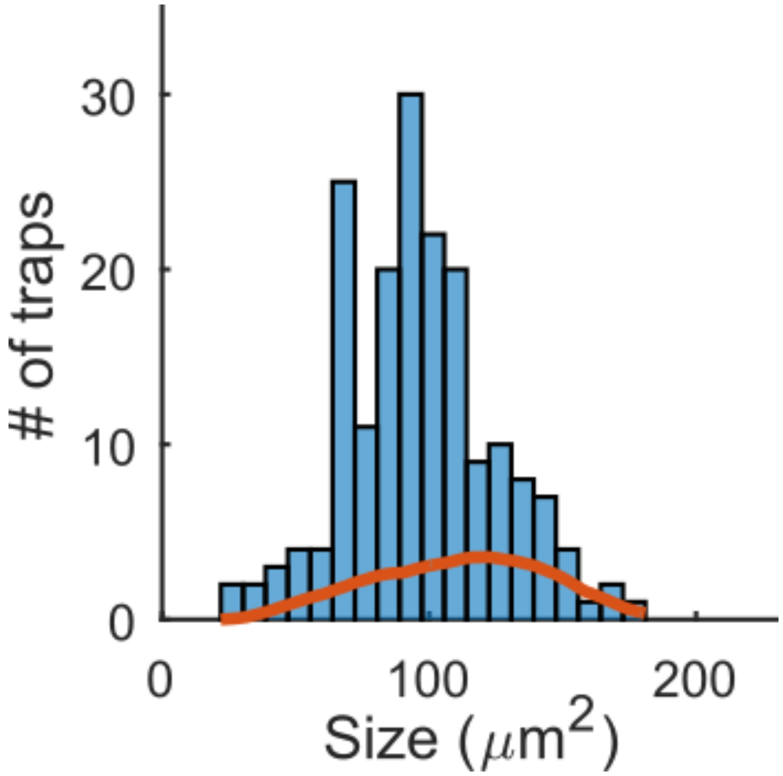

Supplement: Supplementary file 1 — Supplementary Material [file 41598_2018_19483_MOESM1_ESM.pdf]
